# Comparison of nasal microbiota between preterm and full-term infants in early life

**DOI:** 10.1038/s41390-024-03675-6

**Published:** 2024-11-30

**Authors:** Olga Gorlanova, Annika Nissen-Kratzert, Nadja Mostacci, Céline Rüttimann, Noemi Künstle, Andrea Marten, Amanda Gisler, Katharina Bacher, Fabienne Decrue, Yasmin Salem, Jakob Usemann, Insa Korten, Sophie Yammine, Uri Nahum, Sven Schulzke, Philipp Latzin, Martin Röösli, Oliver Fuchs, BILD study consortium, Fiona Beck, Xenia Bovermann, Carmen Casaulta, Marion Curdy, Carla Rebeca Da Silva Sena, Kees de Hoogh, Bettina Frauchiger, Urs Frey, Olga Gorlanova, Léa Kim-Mi Ho Dac, Elisabeth Kieninger, Insa Korten, Noëmi Künstle, Philipp Latzin, Andrea Marten, Loretta Müller, Uri Nahum, Marc-Alexander Oestreich, Martin Röösli, Céline Rüttimann, Sven Schulzke, Pablo Sinues, Ruth Steinberg, Benjamin Stöcklin, Carmen Streibel, Jakob Usemann, Florian Wyler, Sophie Yammine, Markus Hilty, Urs Frey

**Affiliations:** 1 University Children’s Hospital Basel (UKBB), University of Basel, Basel, Switzerland; 2 Division of Pediatric Respiratory Medicine and Allergology, Department of Pediatrics, University Hospital Bern, University of Bern, Bern, Switzerland; 3 Institute for Infectious Diseases, University of Bern, Bern, Switzerland; 4 Centre for Cardiovascular Science, Queen’s Medical Research Institute, Edinburgh, UK; 5 Institute for Medical Engineering and Medical Informatics, University of Applied Sciences and Arts Northwestern Switzerland, Muttenz, Switzerland; 6 Swiss Tropical and Public Health Institute Basel, Allschwil, Switzerland; 7 Priority Research Centre GrowUpWell® and Hunter Medical Research Institute, University of Newcastle, Newcastle, NSW Australia; 8 Department of Biomedical Engineering, University of Basel, Allschwil, Switzerland

## Abstract

**Background:**

The respiratory microbiota influences infant immune system maturation. Little is known about how perinatal, physiological, and environmental exposures impact the nasal microbiota in preterm infants after discharge, or nasal microbiota differences between preterm and healthy full-term infants.

**Methods:**

Nasal swabs (from 136 preterm and 299 full-term infants at mean postmenstrual age of 45 weeks from the prospective Basel-Bern Infant Lung Development cohort) were analyzed by 16S-rRNA gene amplification and sequencing (Illumina MiSeq). Associations were tested with multivariable linear regression and principal coordinate analysis.

**Results:**

Presence of older siblings in preterm infants was associated with β-diversity (PERMANOVA *p* = 0.001) and an increased abundance of *Moraxella* and *Haemophilus*. The nasal microbiota of preterm infants exhibited a distinct composition compared to that of full-term infants (PERMANOVA, *R*^2^ = 0.014, *p* = 0.001), characterized by a reduced abundance of the *Moraxella* and *Dolosigranulum* genera (ANCOM-BC, *p* < 0.05).

**Conclusion:**

Our results indicate that, despite both infant groups having similar nasal microbiota patterns, there are some disparities which suggest that prematurity influences the initial microbiota colonization. In preterm infants the presence of older siblings had an effect on the nasal microbiota, whereas perinatal and early postnatal factors did not show significant effects.

**Impact:**

- Presence of older siblings affected the nasal microbiota of preterm infants.
- This study demonstrated that microbiota composition differs between full-term and preterm infants, with a lower abundance of *Moraxella* and *Dolosigranulum* in preterm infants.
- Examining the differences in nasal microbiota between preterm and full-term infants may contribute to understanding the trajectory of the bacterial component of the nasal microbiota of preterm infants.

## Introduction

The early-life nasal microbiota is important for the development of the immune system and thus an infant’s susceptibility to respiratory infections.^1–5^ The composition and density of the nasal microbiota undergoes notable changes in the first weeks after birth.^6,7^ Its maturation is shaped by complex interactions between host and environmental factors, such as mode of delivery, exposure to other children, type of feeding, viral infections, and exposure to probiotics and antibiotics.^6,8–13^

Most previous studies of the upper respiratory tract microbiota have focused on full-term infants,^1–4,6,8–10^ whereas there are only a few studies that investigate the nasal microbiota in preterm infants with small sample sizes.^14–16^ Bacterial colonization in preterm infants may differ from that in healthy full-term infants because preterm infants are exposed to a variety of interventions in the neonatal intensive care unit (NICU), including postnatal antibiotic treatment, intubation and mechanical ventilation, and prophylactic use of probiotics. Additionally, the NICU environment is associated with the risk of colonization by pathogenic and antimicrobial-resistant bacteria.^14,17,18^ As part of medical treatment, preterm infants receive specialized formula to enhance their growth, potentially influencing microbiota colonization compared to breast milk.^9^ However, there is little evidence available about the nasal microbiota of preterm infants after NICU discharge.

Given their interrupted airway and immune system development, preterm infants are also more susceptible to increased respiratory morbidity in the first year of life.^19^ Moreover, dysbiosis of the microbiota also contributes to the high risk of severe respiratory infection in preterm infants.^16^ For all these reasons, understanding the pattern of nasal microbiota in infants and how it differs from the “optimal” microbiota composition in healthy full-term infants is crucial for development of novel strategies to optimize the establishment of the respiratory microbiota in preterm infants.

In this study, we therefore aimed first, to identify perinatal, physiological, dietary, pharmacological, and environmental exposures associated with the nasal microbiota in preterm infants at mean postmenstrual age of 45 weeks, after hospital discharge, and secondly to compare the microbiota of preterm and full-term infants.

## Methods and materials

### Study design and population

The Basel-Bern Infant Lung Development (BILD) cohort study is an ongoing prospective multicenter study, conducted since 1999 in Bern and since 2011 in Basel, Switzerland (www.bild-cohort.ch), as described previously.^20^ In brief, infants are recruited antenatally or postnatally and followed-up for 12 years or until loss of follow-up. Exclusion criteria were ethnicity other than white Central European, severe congenital anomalies, severe perinatal infections of mother or child, need for ventilation for longer than 3 days in full-term infants, and maternal drug abuse other than smoking.^20^ A signed informed consent from the parents was obtained. The Ethics Committee of Northwest and Central Switzerland (EKNZ, Basel, Switzerland) and the Bernese Cantonal Ethics Research Committee (KEK, Bern, Switzerland) approved the study.

From the overall cohort, a sample of 299 full-term (≥37 weeks of gestation) and 136 preterm infants (<37 weeks of gestation^21^) born between April 2010 and July 2020, with available nasal swabs at mean postmenstrual age of 45 weeks, was included in the present study (Supplementary Material Fig. E1).

### Nasal swabs

Since April 2010, anterior nasal swabs have been obtained by a trained study nurse from all infants at mean postmenstrual age of 45 weeks during a visit to the study clinic. Nasal swabs are collected from both nostrils using 2 flexible, sterile swabs (FLOQSwabs® 516CS01; Copan, Italy) and placed together in a tube with 3 ml medium (UTM-RT^TM^ in Screw-Cap Tube; Copan, Italy). In the laboratory, the medium with nasal secretion is pipetted into micro-screw tubes (Sarstedt; Nürnbrecht, Germany) and then stored in −80 °C freezers until further processing.

The DNA isolation from nasal swabs was conducted by Eurofins Genomics (Germany) using the NucleoSpin Food Kit (Macherey-Nagel; Düren, Germany). Subsequently, the variable V3-V4 regions of the bacterial 16S-rRNA gene were amplified in 25 PCR-cycles using the primer pair 357F/800R. Following amplification, library sequencing was performed.^22^ After quality control, 34 samples with an amplification PCR product concentration below 0.2 ng/µl were excluded. Similarly, negative extraction controls that exhibited insufficient amplification were also excluded from subsequent sequencing.

Next Generation Sequencing (sequencing-by-synthesis) was carried out with equimolar amplicon pools on the MiSeq [300PE] Platform (Illumina; San Diego, CA). More detailed descriptions can be found in the Supplementary Material.

### Data processing

The raw sequencing reads were processed with DADA2 (version 1.18.0).^23^ The forward and reverse reads were trimmed to the length of 280 and 200, respectively. Otherwise, default parameters were used. For assigning taxonomy, Silva database version 138.1^24^ was used.

The raw amplicon sequence variant (ASV) table included a total of 8,999 taxa. First, we removed ASVs with no taxa annotation at the phylum level (*n* = 155) and those with relative abundance <0.1 in all samples using the *phyloseq* package^25^ (v1.34.0). This filtering resulted in an ASV table consisting of 180 taxa, with 4.96% of reads excluded. Next, we removed contaminant ASVs, retaining 172 taxa in total. To identify the contaminant ASVs, the *decontam* R package (“frequency” method) was used.^26^ As metadata, the proxy DNA concentration of the sample was used. This method uses the dependence of the number of counts and the concentration of the input DNA. For the non-contaminant ASVs the number of counts is expected to be independent from the input DNA. For the contaminant ASVs the number of counts is expected to be inversely proportional to input DNA concentration.

### Outcomes

The outcomes of interest were nasal microbiota profile including α-diversity, β-diversity, and relative abundance of phyla, genera, and ASVs. α-diversity of the nasal microbiota describes the richness (the number of taxonomically related groups of bacteria) and evenness (relative abundances of bacteria) of its composition and was assessed using Shannon and Simpson diversity indices. β-diversity is a measure of between-sample diversity that examines the similarities of bacterial community between different comparison groups. α-diversity and β-diversity were calculated using *phyloseq*^25^ with filtered counts and ASVs transformed to relative abundance, respectively.

### Exposure data

The following additional data was obtained either from the birth and hospital records or at the interview: sex (female vs. male), gestational age at birth (weeks), postmenstrual age at date of nasal swab (weeks), postnatal age at date of nasal swab (days), season at date of nasal swab (spring, summer, fall, winter), maternal smoking during pregnancy (yes/no), use of antibiotics in the 3 months prior to birth (yes/no), intrapartum antibiotic prophylaxis (IAP, yes/no), chorioamnionitis (yes/no), mode of delivery (vaginal delivery vs. Cesarean section), any breastfeeding at date of nasal swab (yes/no), presence of older siblings in household (yes/no), hospitalization stay at NICU and hospital room (yes/no), length of hospitalization (calculated as days from birth until discharge), treatments during hospitalization such as intubation (yes/no), oxygen supplementation (yes/no), probiotics use (yes/no), postnatal antibiotic use (yes/no), and the duration of postnatal antibiotic use (days). Prematurity was defined as a gestational age at birth of less than 37 completed weeks.^21^

### Statistical analysis

All analyses were performed in R version 4.0.4 within R studio version 1.4.1717. Continuous data were compared using Student’s *t*-test for data with a normal distribution and the Mann–Whitney test for those with non-normal distribution. Categorical data were compared using Pearson’s chi-square test or Fisher’s exact test, as appropriate.

Primary analysis focused on associations of the microbiota profile with perinatal, physiological, dietary, pharmacological, and environmental exposure factors in preterm infants. The effect of these factors on Shannon and Simpson α-diversity indices was analyzed using a multivariable linear regression model with the adjustment using covariates based on associations from prior studies^10,27,28^ or from our univariable regression model. The multivariable model for preterm infants was adjusted for presence of older siblings, sex, postnatal age, use of antibiotics in the 3 months prior to birth, mode of delivery, probiotics, season, and any breastfeeding at swab collection. For β-diversity, principal coordinate analysis (PCoA) based on the Bray–Curtis distance at the ASV level was used to visualize the variability across different exposure groups. Samples were compared using the permutational multivariable analysis of variance (PERMANOVA)^29^ implemented in the *vegan* package v2.6-2.^30^ The PERMANOVA analysis was adjusted for categorical covariates determined in the previous step. Then, we investigated which genera and ASVs were differentially abundant between exposure factors that showed associations with α-diversity or β-diversity using: (1) Mann–Whitney tests, and (2) Analysis of Compositions of Microbiomes with Bias Correction (ANCOM-BC)^31^ that employs within the linear regression framework and corrects for bias resulting from variations among samples. The ANCOM-BC analysis incorporated the same adjustments as those used for α-diversity models. By default, taxa that feature in less than 10% of all samples are removed from further analysis (other options: p_adj_method = “BH”, zero_cut = 0.9, lib_cut = 0, struc_zero = T, neg_lb = F, tol = 1e-5, max_iter = 100, alpha = 0.05). Multiple testing was accounted for using Benjamini–Hochberg *p*-value correction.

Secondary analysis included the comparison of nasal microbiota between preterm and full-term infants. We examined associations of α-diversity indices with preterm birth in a multivariable linear regression model, accounting for all factors considered for adjustment in the primary analysis. Exposure factors affecting α-diversity indices in full-term infants were assessed using the same approach as for preterm infants. The bacterial communities between preterm and full-term infants were compared using PERMANOVA analysis including all variables selected for adjustment in the α-diversity model except postnatal age. The difference in the relative abundance of phyla, genera, and ASVs between preterm and full-term infants was assessed using Mann–Whitney tests and ANCOM-BC with the same adjustment as those used for the α-diversity model. The significance was defined as Benjamini–Hochberg-adjusted *p*-value_adj_ < 0.05. Additionally, the relative abundance at genus level was visualized using the heatmap available in the microViz R package.^32^

Almost all preterm infants were recruited in Basel, while full-term infants were recruited in both study centers, therefore, we conducted a sensitivity analysis to compare the microbiota in preterm and full-term infants only from Basel. Given that PERMANOVA is a non-parametric alternative to MANOVA, requiring continuous response variables and categorical predictors, we did not adjust the model for postnatal age. Considering the higher variability in postnatal age among preterm infants compared to full-term infants, we assessed the possible correlation of postnatal age with first two principal components for β-diversity in preterm and full-term infants.

## Results

### Population and sample characteristics

Our study population included 136 preterm and 299 full-term infants (Table 1). There was no difference between preterm and full-term infants with respect to sex and maternal smoking during the pregnancy. In contrast to full-term infants, preterms had fewer older siblings, were more frequently exposed to antibiotic use during pregnancy and during the neonatal period, were more frequently born via Cesarean section, and were less breastfed. Although there was a statistically significant difference in postmenstrual age between preterm and full-term infants (mean 44.9 (1.3) weeks vs. 44.6 (1.3) weeks, respectively), the magnitude of this difference was minimal and may not have clinical significance.

**Table 1:** Population characteristics.

	Preterm infants (*N* = 136)	Full-term infants (*N* = 299)	*P*-value
Sex, male *n* (%)	73 (53.7%)	154 (51.5%)	0.751
Gestational age, weeks, mean (SD)	32.4 (2.9)	39.7 (1.1)	<0.001
Postnatal age, days, mean (SD)	85.5 (22.5)	36.0 (6.64)	<0.001
Postmenstrual age at nasal swab, weeks, mean (SD)	44.6 (1.3)	44.9 (1.3)	0.041
Maternal smoking during pregnancy, yes *n* (%)	8 (5.9%)	11 (3.7%)	0.42
Presence of older siblings, yes *n* (%)	44 (32.4%)	170 (56.9%)	<0.001
Season of swab collection			0.258
Winter	39 (28.7%)	67 (22.4%)	
Spring	30 (22.1%)	73 (24.4%)	
Summer	42 (30.9%)	83 (27.8%)	
Fall	25 (18.4%)	76 (25.4%)	
Cesarean section, yes *n* (%)	104 (76.5%)	87 (29.1%)	0.001
Probiotics, yes *n* (%)	55 (40.4%)	1 (0.3%)	0.001
Breastfeeding at date of nasal swab, yes *n* (%)	78 (57.4%)	266 (89.0%)	0.001
Chorioamnionitis, yes *n* (%)	37 (27.2%)	1 (0.3%)	0.001
Antibiotics during pregnancy, yes *n* (%)	60 (44.1%)	73 (24.4%)	<0.001
Antibiotics during last 3 months before birth, yes *n* (%)	51 (37.5%)	35 (11.7%)	<0.001
Intrapartal antibiotic prophylaxis (IAP), yes *n* (%)	96 (70.6%)	80 (26.8%)	<0.001
Postnatal antibiotics for neonatal infection, yes *n* (%)	56 (41.2%)	2 (0.7%)	0.001
Empiric antibiotic, when infection is suspected	43 (76.8%)	2 (100%)	
Neonatal infection without focus	10 (17.9%)		
Early- and late-onset sepsis	3 (5.7%)		
Postnatal antibiotics for neonatal infection, days, mean (SD)	1.7 (2.29)	0.04 (0.4)	<0.001
Hospitalization, yes *n* (%)	115 (84.6%)	1 (0.3%)	0.001
Length of hospitalization, days, mean (SD)	33.5 (27.4)	0.08 (1.39)	<0.001
Intubation, yes *n* (%)	20 (14.7%)	0 (0%)	
Study center, *n* (%)			
Bern	1 (0.7%)	130 (43.5%)	<0.001
Basel	135 (99.3%)	169 (56.5%)	

*P*-values were obtained using t-test, Mann–Whitney test, and Fisher exact test, as appropriate.

After filtering, a median of 99,801 (Q1, Q3: 79,459, 124,781) reads was generated per sample (range = 20,500–665,540). This resulted in an ASV-table containing 172 taxa, represented by four phyla with the relative abundance (expressed as a mean (standard deviation)) of phyla *Firmicutes* (44.1% (33.4)), *Actinobacteriota* (25.5% (29.4)), and *Proteobacteria* (30.4% (35.7)). Less than one percent of ASVs were assigned to *Bacteroidota*.

### Nasal microbiota of preterm infants

**Fig. 1:**
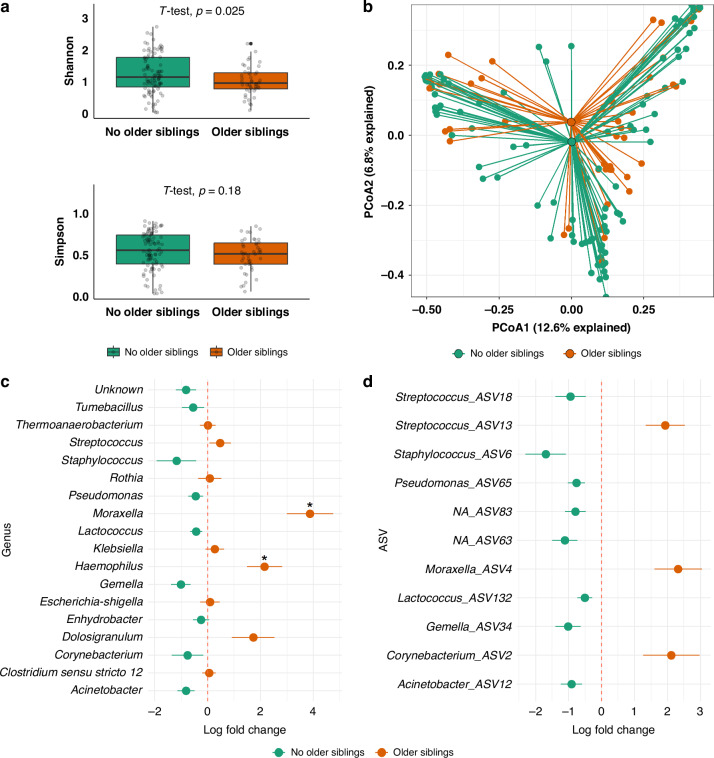
Association between the presence of older siblings and nasal microbiota in preterm infants. **a** Shannon and Simpson diversity in nasal samples of preterm infants with and without older siblings. **b** Principal coordinate analysis (PcoA) of nasal samples (PERMANOVA *p* = 0.013, with adjustment for postnatal antibiotic use, sex, use of antibiotics in the 3 months prior to birth, mode of delivery, probiotics, season of swab collection, and any breastfeeding at swab collection). Differentially enriched genera (**c**) and ASVs (**d**) between infants without older siblings (green) and those with older siblings (orange). Taxa that feature in less than 10% of all samples were removed from analysis. Points show the log fold change as given by ANCOM-BC, error bars denote the standard error. The analysis was adjusted for postnatal antibiotic use, sex, postnatal age, use of antibiotics in the 3 months prior to birth, mode of delivery, probiotics, season of swab collection, and any breastfeeding at swab collection. ASVs (**d**) reaching the nominal significance are shown. Asterisks indicate significant *p*-values following Benjamini–Hochberg adjustment for multiple testing.

In the univariable model, preterm infants with older siblings had a significantly lower Shannon diversity index compared to those without (Supplementary Material Tables E1 and E2, Fig. 1a). However, after adjustment for risk factors, this association was not significant (Supplementary Material Tables E3 and E4).

For β-diversity, when we assessed the difference between exposure groups based on the Bray–Curtis distance, we found a difference depending on presence of older siblings (PERMANOVA *p* = 0.013, Fig. 1b). The measure of group separation (*R*^*2*^ statistics varying from 0—no separation to 1—total separation) for presence of older siblings was relatively small (*R*^*2*^ = 0.017, online Supplementary Table E5). Other exposure factors were not associated with microbial composition assessed by α- and β-diversity indices in the adjusted analysis (Supplementary Material Tables E3–E5).

At the phylum level, infants with older siblings had a higher abundance of *Actinobacteriota* than infants without older siblings (*p* = 0.016, Supplementary Material Fig. E2). Characteristics of the relative abundance of genera in the nasal microbiota of preterm infants by the presence of older siblings are presented in Fig. E2 and Table E6. ANCOM-BC analysis revealed that the presence of older siblings was positively associated with *Haemophilus* and *Moraxella* abundance (*p*-value_adj_ = 0.022 and *p*-value_adj_ = 0.0001, respectively, Fig. 1c). No specific ASV was found to be associated with the presence of older siblings in preterm infants (Fig. 1d). ANCOM-BC analysis further revealed that the probiotic use was negatively associated with *Dolosigranulum* abundance (*p*-value_adj_ = 0.0035, respectively, Fig. E3, Table E7). Lower probiotic-associated ASVs included genera *Dolosigranulum*, *Tumebacillus* and a genus without taxa annotation (Fig. E3). Infants with and without probiotic use differed significantly in population characteristics: those who received probiotics had lower gestational age and birth weight, were predominantly born by Cesarean section, and more frequently received postnatal antibiotics (Table E8).

### Difference in nasal microbiota between preterm and full-term infants

**Fig. 2:**
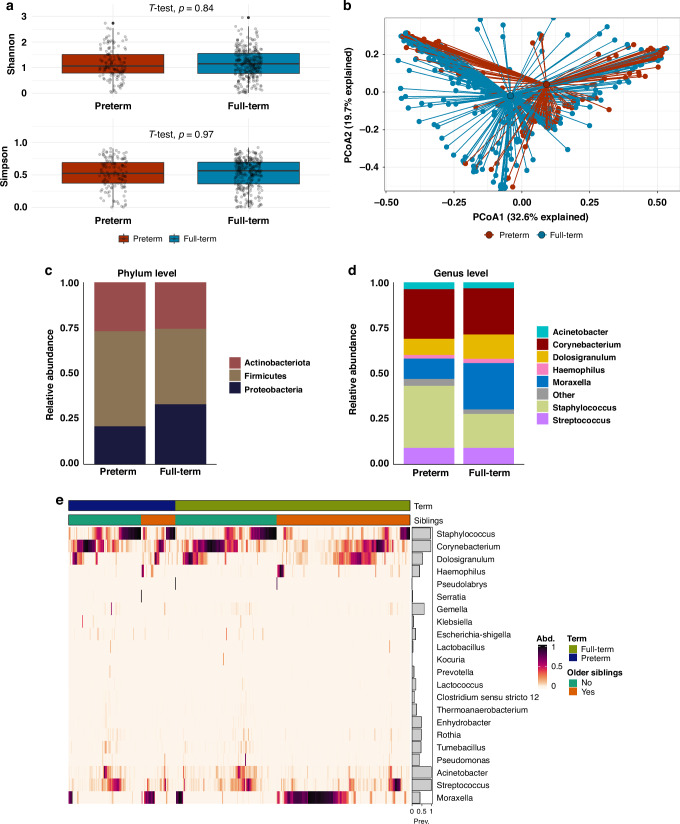
Nasal microbiota in preterm and full-term infants. **a** Shannon and Simpson α-diversity in nasal samples of preterm and full-term infants. **b** Principal coordinate analysis (PcoA) of nasal samples of preterm and full-term infants (PERMANOVA *p* = 0.001, with adjustment for presence of older siblings, sex, use of antibiotics in the 3 months prior to birth, postnatal antibiotic use, probiotics, mode of delivery, season of swab collection, any breastfeeding at swab collection, and study center). Bar plots show average relative abundances of phyla (**c**) and genera (**d**) level in preterm and full-term infants. Low abundant genera with a mean below 0.01 were grouped into the “other” category. The low abundant phylum *Bacteroidota* was not included due to its mean abundance being less than 0.01. **(e)** Heatmap summarizing the relative abundance at genus level and sorted by full-term birth and presence of older siblings.

There were no significant differences in any of the measured α-diversity indices between preterm and full-term infants (Fig. 2a, Supplementary Material Table E9). In contrast, there was a significant difference in the overall community structure of the nasal microbiota between preterm and full-term infants (PERMANOVA *p* = 0.001, *R*^*2*^ = 0.014, Fig. 2b and Table E10).

**Fig. 3:**
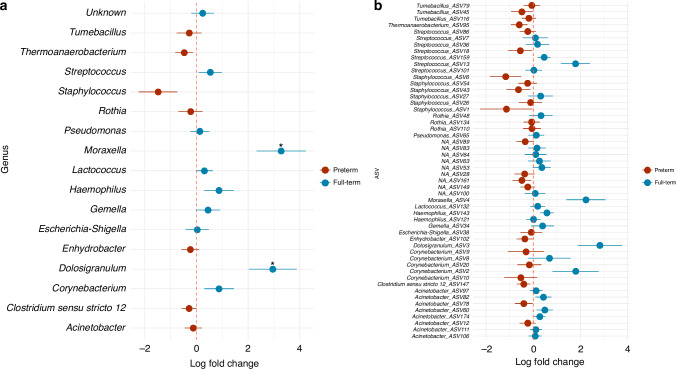
**Differentially abundant genera and ASVs in nasal samples of preterm and full-term infants**. Differential abundance testing was done using ANCOM-BC accounting for postnatal age, presence of older siblings, season, use of antibiotics in the 3 months prior to birth, postnatal antibiotic use, breastfeeding at swab collection, and study center. Taxa that feature in less than 10% of all samples were removed from analysis. Points show the log fold change as given by ANCOM-BC, error bars denote the standard error at the genus (**a**) and ASV (**b**) level of taxa in preterm and full-term infants. Asterisks indicate significant *p*-values following Benjamini–Hochberg adjustment for multiple testing.

The nasal microbiota of preterm infants had a lower relative abundance of Proteobacteria on average compared with full-term infants (Fig. 2c, Supplementary Material Table E11). At the genus level, preterm infants’ nasal microbiota had a higher relative abundance of *Staphylococcus* and *Pseudomonas*, and a lower relative abundance of *Moraxella*, *Dolosigranulum*, and *Haemophilus* compared to full-term infants (Mann–Whitney test, *p*-value_adj_ < 0.05, Table E12). However, in the adjusted model, only Moraxella and Dolosigranulum were found to be significantly differential in abundance (ANCOM-BC, *p*-value_adj_ = 0.010 and *p*-value_adj_ = 0.024, respectively, Fig. 3a). Notably, full-term infants with older siblings exhibited a higher abundance of *Moraxella* compared to preterm infants with older siblings (Fig. 2e, Mann–Whitney test *p* = 0.015). Furthermore, there was no particular ASV that was differentially abundant between preterm and full-term infants in the adjusted ANCOM-BC analysis (Fig. 3b).

In a sensitivity analysis we assessed the impact of inter-center difference on the association of prematurity with the nasal microbiota, restricting our sample to the Basel study center. Associations remained similar (Supplementary Material Fig. E4). We observed no correlation between postnatal age and PCoA1 or PCoA2 in both preterm and full-term infants (Fig. E5).

## Discussion

To our knowledge, this is the first study to investigate the exposure factors associated with nasal microbiota in preterm infants after hospital discharge in a large, healthy cohort sharing the same developmental stage (postmenstrual age) and home environment prior to the first viral infection. Our findings suggest that β-diversity measure and relative abundance were affected by the presence of older siblings, but not other exposure factors. Moreover, we compared the nasal microbiota between preterm and healthy full-term infants at mean postmenstrual age of 45 weeks and found significant differences in β-diversity and the relative abundance of different bacterial taxa. Preterm infants showed a lower abundance of *Moraxella* and the health-associated commensal *Dolosigranulum* species compared to full-term infants.

In contrast to previous research that emphasized the impact of perinatal and early postnatal factors on nasal microbiota in preterm infants (including the NICU environment, mode of delivery, and postnatal antibiotic use), we observed no such effects in our study. For instance, previous studies suggested that the NICU environment might alter the microbiota of preterm infants.^33^ Khamash et al.^15^ were able to demonstrate colonization with *Staphylococcus aureus* especially in hospitalized preterm infants aged 17–59 days with an initially significantly lower richness. They concluded that this may indicate that neonates with lower richness are predisposed to *Staphylococcus aureus* dominance or that *Staphylococcus aureus* dominance reduces co-colonization with health-associated commensals.^15^ However, in our study, hospitalization per se had no independent influence on nasal microbiota, when adjusted for other risk factors. This discrepancy may be attributed to the fact that, by postmenstrual age of 45 weeks, the effects of perinatal and early postnatal factors had diminished.

In our study, the nasal microbiota in preterm infants was associated with the presence of older siblings. While research on respiratory microbiota is limited and predominantly conducted in full-term infants, our findings generally align with previous studies. For example, Hasegawa et al.^11^ and Christensen et al.^34^ observed increased abundance of *Moraxella* in full-term infants. This suggests a potential transfer of this specific nasal microbiota pattern from siblings, potentially associated with a higher infection risk due to the presence of older siblings. It has been well-described that a Moraxella-dominant profile is associated with a higher risk of respiratory infection in the first 2 years of life.^27^

We also found that the abundance of *Dolosigranulum* was lower in the group of preterm infants who received probiotics. *Dolosigranulum* is an important age-discriminatory genus that increases with age over the first 2 months of life;^2^ its reduction has been associated with antibiotic treatment^18^ and Cesarean section.^2^ Probiotic administration depended on gestational age and weight in our cohort. Typically, preterm infants with a gestational age of <32 weeks or <1500 g birth weight, or both, received the probiotic Infloran. Moreover, probiotic use was strongly associated with more frequent postnatal antibiotic use, Cesarean section births, and an older postnatal age at the time of swab collection compared to infants not receiving probiotics. Thus, the observed negative association between probiotic use and Dolosigranulum abundance may reflect collinearity with these variables. The difference in nasal microbiota between preterm and full-term infants might be partly explained in the context of existing literature on known influencing factors in early life.^4,6,9^ Since preterm infants significantly differ in development during the first few weeks (e.g., immune development), we suspect that the immaturity of the preterm infants may add to the differences in microbiota composition compared to full-term infants.

In typical scenarios, the neonatal period sees a transformation in the microbiota of the respiratory tract, transitioning from a prevalence of *Staphylococcus* to a predominance of *Corynebacterium* or *Dolosigranulum*, and eventually to an abundance of *Moraxella* at 1.5 months of age.^2^ Additionally, these shifting trends appear to correlate with infant feeding habits, presence of older siblings, season of birth, and the frequency of respiratory infections in the first 2 years of life.^2,6,9^ In our study, we found that the nasal microbiota of both preterm and full-term infants was predominantly colonized by four genera: *Staphylococcus, Moraxella, Corynebacterium,* and *Dolosigranulum*. However, preterm infants had a higher abundance of *Staphylococcus* alongside lower levels of *Moraxella* and *Dolosigranulum*. This difference may be attributed to the influence of the NICU environment on nasal microbiota development in preterm infants, which could lead to a higher prevalence of *Staphylococcus*. Additionally, since preterm infants have limited or no contact with older siblings during hospitalization, the transition from *Staphylococcus* to *Corynebacterium* or *Dolosigranulum*, and finally to an abundance of *Moraxella*, may be delayed. Furthermore, the difference in *Moraxella* abundance might also be partially attributed to the fact that full-term infants with older siblings exhibited a higher abundance of *Moraxella* compared to preterm infants with older siblings.

Preterm infants are typically more often born by Cesarean section; however, we found no association between the nasal microbiota and mode of delivery in preterm infants. This is in-line with other studies, which showed that the difference in microbiota based on the mode of delivery disappeared^35^ or significantly decreased^8^ over the first weeks of life. However, we did observe significant dissimilarity in β-diversity between vaginally born and Cesarean-section-born full-term infants, as well as when preterm and full-term infants were combined, but not in preterm infants. This may be attributed to reduced power due to the smaller sample size, or it may indicate that postnatal age and other factors such as probiotic use could modify the effect of mode of delivery on β-diversity in preterm infants.

In our study, preterm infants were breastfed less frequently compared to full-term infants and were more likely to receive antibiotics. Interestingly, we did not observe a significant effect of breastfeeding on the nasal microbiota in either preterm or full-term infants. This contrasts with findings from Biesbroek et al.^36^ who reported that exclusively breastfed full-term infants had a *Corynebacterium*- and *Dolosigranulum*-dominated nasal microbiota at 6 weeks of age, with a concurrent negative association with *Staphylococcus aureus* colonization. This difference may be partly attributed to the very high proportion of breastfed full-term infants in our cohort. However, it is important to note that the influence of feeding type on the microbiota of preterm infants has been studied only in the gut so far, and results were inconsistent.^37,38^

The strengths of our study are the sample sizes with a high proportion of preterm infants, and the prospective design involving the collection of a wide range of factors that could possibly influence the nasal microbiota. In addition, a high qualitative standard could be maintained throughout the entire period, as all swabs were taken by trained personnel according to a uniform protocol. However, since the BILD cohort study is primarily concerned with lung development in childhood, preterm and full-term children are followed at the same postmenstrual age in order to be able to compare the effect of prematurity on lung development. While this study design is cross-sectional in nature, it allows comparison with a postmenstrual-age-matched large control group, however this is a single observation at a given time point. Since developmental trends and variations are to be expected, future studies may need to look at the longitudinal development in the nasal microbiota in both groups of preterm and full-term infants. The human microbiota begins to develop at birth and thus from day one of life and is influenced by external factors.^8,39^ Nevertheless, no effect of postnatal age or hospital stay on the microbiota diversity was found in preterm infants. Therefore, depending on the degree of prematurity, some of the preterm infants in our study were significantly older in terms of days of life than the full-term infants. Possible inter-center differences were elucidated in detail since the number of preterm infants in Basel was higher than in Bern. Sensitivity analysis excluding infants from Bern replicated findings from the whole cohort. Finally, generalization of our findings can only be made with caution because the study infants were drawn from the Central European population, which may not be generalizable to other ethnicities and geographically different locations.

## Conclusion

In this study, we found that the presence of older siblings was associated with a difference in nasal microbiota composition. However, other perinatal and early postnatal factors did not demonstrate significant effects on the nasal microbiota. Additionally, differences in the nasal microbiota were observed between preterm infants and full-term infants at postmenstrual age of 45 weeks, as assessed by the Bray–Curtis β-diversity index and the relative abundance. These differences are small and might be partially explained by the fact that preterm infants are exposed to different perinatal, physiological, treatment, and environmental factors and that none of these exposure factors work in isolation. Furthermore, when compared to other nasal microbiota studies conducted shortly after birth in the hospital environment, the differences between preterm and full-term infants are relatively small at the postmenstrual age of 45 weeks in the home environment, which indicates the convergence of nasal microbiota in preterm and full-term infants. This finding may be relevant for the better understanding of the baseline starting conditions for airway microbiota development and its role for subsequent disease development in preterm infants in their natural home environment.

## Supplementary information

Supplementary Material

Table_E7_CLEAN

## Data Availability

The 16S amplicon data has been submitted to the NCBI SRA database (https://www.ncbi.nlm.nih.gov/bioproject), under accession number PRJNA944094. Deidentified individual data (including data dictionaries) will be made available from the corresponding author upon request with study proposal and completion of a satisfactory data transfer agreement. Proposals should be submitted to urs.frey@ukbb.ch.

## References

[1] Bisgaard, H. et al. Childhood asthma after bacterial colonization of the airway in neonates. *N. Engl. J. Med.* **357**, 1487–1495 (2007). 17928596 10.1056/NEJMoa052632

[2] Biesbroek, G. et al. Early respiratory microbiota composition determines bacterial succession patterns and respiratory health in children. *Am. J. Respir. Crit. Care Med.* **190**, 1283–1292 (2014). 25329446 10.1164/rccm.201407-1240OC

[3] Teo, S. M. et al. The infant nasopharyngeal microbiome impacts severity of lower respiratory infection and risk of asthma development. *Cell Host Microbe* **17**, 704–715 (2015). 25865368 10.1016/j.chom.2015.03.008 PMC4433433

[4] Toivonen, L. et al. Early nasal microbiota and acute respiratory infections during the first years of life. *Thorax* **74**, 592–599 (2019). 31076501 10.1136/thoraxjnl-2018-212629

[5] Vissing, N. H., Chawes, B. L. & Bisgaard, H. Increased risk of pneumonia and bronchiolitis after bacterial colonization of the airways as neonates. *Am. J. Respir. Crit. Care Med.* **188**, 1246–1252 (2013). 24090102 10.1164/rccm.201302-0215OC

[6] Mika, M. et al. Dynamics of the nasal microbiota in infancy: a prospective cohort study. *J. Allergy Clin. Immunol.* **135**, 905–912. e911 (2015). 25636948 10.1016/j.jaci.2014.12.1909

[7] Salter, S. J. et al. A longitudinal study of the infant nasopharyngeal microbiota: the effects of age, illness and antibiotic use in a cohort of South East Asian children. *PLoS Negl. Trop. Dis.* **11**, e0005975 (2017). 28968382 10.1371/journal.pntd.0005975 PMC5638608

[8] Bosch, A. A. et al. Development of upper respiratory tract microbiota in infancy is affected by mode of delivery. *EBioMedicine* **9**, 336–345 (2016). 27333043 10.1016/j.ebiom.2016.05.031 PMC4972531

[9] Bosch, A. A. et al. Maturation of the infant respiratory microbiota, environmental drivers, and health consequences. a prospective cohort study. *Am. J. Respir. Crit. Care Med.* **196**, 1582–1590 (2017). 28665684 10.1164/rccm.201703-0554OC

[10] Korten, I. et al. Interactions of respiratory viruses and the nasal microbiota during the first year of life in healthy infants. *mSphere* **1**, e00312–00316 (2016). 27904883 10.1128/mSphere.00312-16 PMC5120172

[11] Hasegawa, K. et al. Household siblings and nasal and fecal microbiota in infants. *Pediatr. Int.* **59**, 473–481 (2017). 27638139 10.1111/ped.13168 PMC5354996

[12] Bogaert, D. et al. Variability and diversity of nasopharyngeal microbiota in children: a metagenomic analysis. *PloS ONE* **6**, e17035 (2011). 21386965 10.1371/journal.pone.0017035 PMC3046172

[13] Ray, K. J., Santee, C., McCauley, K., Panzer, A. R. & Lynch, S. V. Gut bifidobacteria enrichment following oral lactobacillus-supplementation is associated with clinical improvements in children with cystic fibrosis. *BMC Pulm. Med.* **22**, 1–9 (2022). 35902830 10.1186/s12890-022-02078-9 PMC9330662

[14] Cason, C. et al. Microbial contamination in hospital environment has the potential to colonize preterm newborns’ nasal cavities. *Pathogens* **10**, 615 (2021). 34067889 10.3390/pathogens10050615 PMC8156200

[15] Khamash, D. F. et al. The association between the developing nasal microbiota of hospitalized neonates and Staphylococcus aureus colonization. *Open Forum Infect. Dis.* **6**, ofz062 (2019). 30949531 10.1093/ofid/ofz062 PMC6441571

[16] Perez, G. F. et al. Nasopharyngeal Microbiome in premature infants and stability during rhinovirus infection. *J. Investig. Med.* **65**, 984–990 (2017). 28363939 10.1136/jim-2017-000414 PMC5534185

[17] Mourani, P. M., Harris, J. K., Sontag, M. K., Robertson, C. E. & Abman, S. H. Molecular identification of bacteria in tracheal aspirate fluid from mechanically ventilated preterm infants. *PloS ONE* **6**, e25959 (2011). 22016793 10.1371/journal.pone.0025959 PMC3189942

[18] Raita, Y. et al. Maturation of nasal microbiota and antibiotic exposures during early childhood: a population-based cohort study. *Clin. Microbiol. Infect.* **27**, 283.e281–283.e287 (2021). 10.1016/j.cmi.2020.05.033 32505584

[19] Collins, A., Weitkamp, J.-H. & Wynn, J. L. Why are preterm newborns at increased risk of infection? *Arch. Dis. Child. Fetal Neonatal Ed.* **103**, F391–F394 (2018). 29382648 10.1136/archdischild-2017-313595 PMC6013388

[20] Fuchs, O., Latzin, P., Kuehni, C. E. & Frey, U. Cohort profile: the Bern infant lung development cohort. *Int. J. Epidemiol.* **41**, 366–376 (2012). 21233140 10.1093/ije/dyq239 PMC7108546

[21] World Health Organization (WHO). International Statistical Classification of Diseases and Related Health Problems, accessed 9 September 2020. Available from: https://icd.who.int/browse10/2019/en#/P07.3.

[22] Hilty, M. et al. Nasopharyngeal microbiota in infants with acute otitis media. *J. Infect. Dis.* **205**, 1048–1055 (2012). 22351941 10.1093/infdis/jis024 PMC7107284

[23] Callahan, B. J. et al. Dada2: high-resolution sample inference from illumina amplicon data. *Nat. methods* **13**, 581–583 (2016). 27214047 10.1038/nmeth.3869 PMC4927377

[24] Yarza, P. et al. Uniting the classification of cultured and uncultured bacteria and archaea using 16s rRNA gene sequences. *Nat. Rev. Microbiol.* **12**, 635–645 (2014). 25118885 10.1038/nrmicro3330

[25] McMurdie, P. J. & Holmes, S. Phyloseq: an R package for reproducible interactive analysis and graphics of microbiome census data. *PloS ONE* **8**, e61217 (2013). 23630581 10.1371/journal.pone.0061217 PMC3632530

[26] Davis, N. M., Proctor, D. M., Holmes, S. P., Relman, D. A. & Callahan, B. J. Simple statistical identification and removal of contaminant sequences in marker-gene and metagenomics data. *Microbiome* **6**, 1–14 (2018). 30558668 10.1186/s40168-018-0605-2 PMC6298009

[27] Toivonen, L. et al. Antibiotic treatments during infancy, changes in nasal microbiota, and asthma development: population-based cohort study. *Clin. Infect. Dis.* **72**, 1546–1554 (2021). 32170305 10.1093/cid/ciaa262 PMC8096219

[28] Chen, J. et al. Sex differences in gut microbial development of preterm infant twins in early life: a longitudinal analysis. *Front. Cell. Infect. Microbiol.* **11**, 671074 (2021). 10.3389/fcimb.2021.671074 PMC8387566 34458157

[29] Anderson, M. J. A new method for non‐parametric multivariate analysis of variance. *Austral Ecol.* **26**, 32–46 (2001).

[30] Oksanen, J. et al. Package ‘Vegan’. *Community ecology package, version* 2 (2013).

[31] Lin, H. & Peddada, S. D. Analysis of compositions of microbiomes with bias correction. *Nat. Commun.* **11**, 3514 (2020). 32665548 10.1038/s41467-020-17041-7 PMC7360769

[32] Barnett, D. J., Arts, I. C. & Penders, J. Microviz: an R package for microbiome data visualization and statistics. *J. Open Source Softw.* **6**, 3201 (2021).

[33] Hartz, L. E., Bradshaw, W. & Brandon, D. H. Potential NICU environmental influences on the neonate’s microbiome: a systematic review. *Adv. Neonatal Care* **15**, 324 (2015). 26340035 10.1097/ANC.0000000000000220 PMC4583357

[34] Christensen, E. D. et al. The developing airway and gut microbiota in early life is influenced by age of older siblings. *Microbiome* **10**, 106 (2022). 35831879 10.1186/s40168-022-01305-z PMC9277889

[35] Chu, D. M. et al. Maturation of the infant microbiome community structure and function across multiple body sites and in relation to mode of delivery. *Nat. Med.* **23**, 314–326 (2017). 28112736 10.1038/nm.4272 PMC5345907

[36] Biesbroek, G. et al. The impact of breastfeeding on nasopharyngeal microbial communities in infants. *Am. J. Respir. Crit. Care Med.* **190**, 298–308 (2014). 24921688 10.1164/rccm.201401-0073OC

[37] Gregory, K. E. et al. Influence of maternal breast milk ingestion on acquisition of the intestinal microbiome in preterm infants. *Microbiome* **4**, 1–15 (2016). 28034306 10.1186/s40168-016-0214-x PMC5200970

[38] Dahl, C. et al. Preterm infants have distinct microbiomes not explained by mode of delivery, breastfeeding duration or antibiotic exposure. *Int. J. Epidemiol.* **47**, 1658–1669 (2018). 29688458 10.1093/ije/dyy064

[39] Dominguez-Bello, M. G. et al. Delivery mode shapes the acquisition and structure of the initial microbiota across multiple body habitats in newborns. *Proc. Natl. Acad. Sci. USA* **107**, 11971–11975 (2010). 20566857 10.1073/pnas.1002601107 PMC2900693

