# Supplementary material for: Comparison of nasal microbiota between preterm and full-term infants in early life
Source: Pediatr Res. 2024 Nov 30;98(2):636–44. doi: 10.1038/s41390-024-03675-6 (PMC12454110; doi:10.1038/s41390-024-03675-6)
Supplement: Supplementary file 1 — Supplementary Material [file 41390_2024_3675_MOESM1_ESM.pdf]

Supplementary Material

Comparison of nasal microbiota between preterm and full-term infants in early life

Olga Gorlanova[1,2]*, Annika Nissen-Kratzert[1,2]*, Nadja Mostacci[3], Céline Rüttimann[1,2], Noemi Künstle[1,2], Andrea Marten[1,2], Amanda Gisler[1,2], Katharina Bacher[3], Fabienne Decrue[1,4], Yasmin Salem[1,2], Jakob Usemann[1,2], Insa Korten[2], Sophie Yammine[2,2], Uri Nahum[1,2], Sven Schulzke[1,2], Philipp Latzin[2,1], Martin Röösli[5], Oliver Fuchs[2], BILD study consortium[†], Markus Hilty[3]*, and Urs Frey[1, 2]*

[1] University Children's Hospital Basel (UKBB), University of Basel, Basel, Switzerland

[2] Division of Pediatric Respiratory Medicine and Allergology, Department of Pediatrics, University Hospital Bern, University of Bern, Switzerland

[3] Institute for Infectious Diseases, University of Bern, Bern, Switzerland

[4] Centre for Cardiovascular Science, Queen's Medical Research Institute, Edinburgh, EH4 3RL, United Kingdom

[5] Swiss Tropical and Public Health Institute Basel, Allschwil, Switzerland

† A list of authors and their affiliations appears at the end of the main maunscript

[*] These authors contributed equally

Corresponding author:

Prof. Urs Frey, University Children's Hospital Basel (UKBB), Switzerland

Spitalstrasse 33, 4056 Basel, Switzerland

Email: urs.frey@ukbb.ch

CONTENTS

METHODS 3

References 6

TABLES 7

Table E1: Univariable linear regression model of Shannon diversity in preterm and full-term infants 7

Table E2: Univariable linear regression model of richness in preterm and full-term infants 8

Table E3: Multivariable linear regression model of Shannon indices in preterm and full-term infants 9

Table E4: Multivariable linear regression model of Simpson indices in preterm and full-term infants 10

Table E5: Effect of exposure factors on β-diversity (Bray–Curtis distance) of preterm infants by permutational multivariate analysis of variance (PERMANOVA) 11

Table E6: Relative abundance at genus level in preterm infants stratified by presence of older siblings 12

Table E8: Population characteristics of preterm infants stratified by probiotic use 13

Table E9: Multivariable linear regression model of Shannon diversity and Simpson indices in all infants 14

Table E10: Effect of exposure factors on β-diversity (Bray–Curtis distance) in full-term infants and the overall population preterm and full-term infants using permutational multivariate analysis of variance (PERMANOVA) 15

Table E11: Relative abundance at phylum level in preterm and full-term infants 16

Table E12: Relative abundance at genus level in preterm and full-term infants 17

FIGURES 18

Figure E1: Study flowchart 18

Figure E2: Mean of relative abundance of the phyla (**a**) and genera (**b**) identified in groups of preterm infants with and without older siblings. 19

Figure E3: Differentially abundant genera and ASVs in nasal samples of preterm infants with and without probiotic use. 20

Figure E4: The nasal microbiota in preterm and full-term infants only from Basel (n=304). 21

Figure E5: Scatter plot illustrating the relationship between postnatal age and the first two principal components (PCs) for β-diversity in preterm and full-term infants. 23

METHODS
Study Design and Study Population

The Basel-Bern Infant Lung Development (BILD) Study is a prospective cohort study that has been ongoing since 1999 and aims to investigate the impact of environmental factors on the lung development. Special aspects of causal research are the assessment of the influence of environmental factors (indoor and outdoor air pollution), viral infections and genetic predisposing factors on lung development, as well as the development of the immune system and later respiratory symptoms. Increasing attention is being paid to at-risk children (e.g., premature infants or children with asthmatic mothers) and their possible increased risk of developing respiratory diseases and asthma in particular.

Participants, respectively their parents, are recruited prenatally or at the latest three weeks after birth at two Swiss university hospitals (Department of Gynaecology and Obstetrics at Inselspital, Bern University Hospital and University Hospital of Basel). White Central European, healthy full-term (≥37 weeks of gestation[1]) or preterm infants (<37 weeks of gestation[1]), as well as preterm infants with chronic lung diseases, are included. Exclusion criteria are severe congenital anomalies, severe perinatal infections of mother or infant, need for ventilation for more than three days in full-term infants, maternal drug abuse except smoking, and language barriers. More details about the Basel-Bern Infant Lung Development (BILD) cohort study and the entire study process are described elsewhere.[2] In total, more than 1000 children have already been included in the BILD cohort study.

Since April 2010, anterior nasal swabs are taken from all infants for the microbiological examination of bacterial flora. Swabs are performed at the study centre during the visit for lung function measurements at post-menstrual age of 44–46 weeks in preterm and 4–6 weeks after birth in term infants. In this study, between April 2010 and July 2020 we could include a subpopulation of 475 infants with nasal swabs at mean post-menstrual age of 45 weeks.

A signed informed consent from at least one parent is provided. No financial compensation is paid to the study participants or the parents. The Ethics Committee of the Canton of Bern and Basel approved to this study.

Data Collection

For the present study, anterior nasal swabs were taken between April 2010 and July 2020. All participants who visited the study centre for lung function measurements at mean post-menstrual age of 45 weeks took part. Trained study nurses collected anterior nasal swabs from both nostrils using 2 flexible, sterile swabs (FLOQSwabs® 516CS01, Copan; Italy). The swabs were inserted into the nostrils until the entire padded part was in contact with the nasal mucosa. The swabs were moved along the mucosa several times in circular movements. Then both swabs were placed together in a tube with 3ml stabilizing medium (UTM-RT™ in Screw-Cap Tube, Copan; Italy). The tubes were stored at room temperature for about 2–5 hours. Before further processing in the laboratory, the tubes with medium and nasal secretion were shaken. The solution was divided into 3 aliquots of 1ml each using an Eppendorf pipette and placed into micro-screw tubes (Sarstedt; Nürnbrecht, Germany). Until further processing they were kept frozen at -80°C.

Bacterial DNA extraction

The construction of the phylogenetic library was performed after isolation of bacterial DNA, amplification of the variable regions (V3 to V4) of the bacterial 16S-rRNA gene, and next generation sequencing.

Initially, pilot 16S amplification was performed in-house for several samples. However, due to excessive dilution with medium, the bacterial density was too low in the majority of the samples, resulting in their exclusion from further analysis. Subsequently, we modified our approach by centrifuging the initial solution to concentrate the bacterial DNA, resulting in a

residual amount of 200µl for several other samples. Following this adjustment, these samples exhibited a significantly higher DNA concentration after 16S rRNA amplification.

To ensure consistency in the methods, the protocol for increasing DNA concentration prior to 16S amplification was sent with the remaining samples to Eurofins Genomics (Germany), the commercial provider for complete processing. There, after thawing, all samples were centrifuged at 12000g for 5 minutes. The supernatant was discarded except for the required 200ul. The subsequent steps of DNA extraction were performed using the NucleoSpin Food Kit (Macherey-Nagel; Düren, Germany). The pellets including 200ul supernatant were mixed with 200ul LysisBuffer CF (preheated to 65°C) and pre-texted. Finally, another 350µl of LysisBuffer CF (preheated to 65°C) was added, mixed and 10µl of Proteinase K was added. The samples were incubated overnight at 65°C and then further processed according to the Macherey-Nagel protocol.

16S-rRNA Amplification and Next Generation Sequencing

The extracted DNA was further processed by 16S-rRNA amplification.

In the present study the primer pair 357F (TACGGGAGGCAGCAG)/800R (CCAGGGTATCTAATCC) was used for the amplification of the hypervariable V3 and V4 regions of the bacterial 16S-rRNA genes. Each sample has passed through 25 PCR cycles. More detailed descriptions of 16S-rRNA amplification can be found elsewhere [3].

Next generation sequencing (sequencing by synthesis) was carried out on the MiSeq [300PE] Platform (Illumina; San Diego, CA).

Internal company protocols and manufacturer’s specifications were used for 16S-rRNA amplification and next generation sequencing.

Quality control

Due to known low bacterial density in nasal swabs in newborns[4] different quality controls were performed to control for possible contamination. Company-owned extraction controls were analysed along with the samples and each showed no sequenceable material and thus no contamination. Samples with DNA concentration less than 0.2ng/µl after PCR were excluded for microbiota analysis, because the lower the bacterial density of a sample, the greater the possible influence of contamination. Therefore, we excluded 34 samples from the study with a PCR product concentration below 0.2ng/µl after 25 PCR cycles.

Due to these findings no co-clustering with DNA blanks was possible or necessary. Four samples had to be excluded due to lack of quality standards, as the samples were taken by the parents at home. One sample had to be ruled out due to missing clinical information and another due to a viral infection at date of nasal swab.

References

1 World Health Organization (WHO). International Statistical Classification of Diseases and Related Health Problems. **Available from: https://icd.who.int/browse10/2019/en#/P07.3.** (ICD-10. 2019 [accessed 2020 Sep 9]).

2 Fuchs, O., Latzin, P., Kuehni, C. E. & Frey, U. Cohort Profile: The Bern Infant Lung Development Cohort. *International journal of epidemiology* **41**, 366-376 (2012).

3 Hilty, M. et al. Nasopharyngeal Microbiota in Infants with Acute Otitis Media. *Journal of infectious diseases* **205**, 1048-1055 (2012).

4 Biesbroek, G. et al. Deep Sequencing Analyses of Low Density Microbial Communities: Working at the Boundary of Accurate Microbiota Detection. *PloS one* **7**, e32942 (2012).

TABLES

Table E1: Univariable linear regression model of Shannon diversity in preterm and full-term infants

	Preterm infants			Full-term infants		
Variables	***β***	***95% CI***	***p-value***	***β***	***95% CI***	***p-value***
Gestational age, weeks	0.00	-0.04; 0.03	0.899	0.03	-0.02; 0.09	0.241
Postmenstrual age at nasal swab, weeks	0.01	-0.07; 0.09	0.822	0.04	-0.01; 0.09	0.106
Postnatal age, weeks	0.00	0; 0	0.837	0.00	-0.01; 0.01	0.432
Cesarean section, yes	0.07	-0.16; 0.31	0.546	0.01	-0.13; 0.15	0.871
Smoking during pregnancy, yes	-0.12	-0.55; 0.3	0.571	-0.05	-0.39; 0.29	0.761
Antibiotics during pregnancy, yes	-0.10	-0.3; 0.1	0.320	-0.15	-0.3; 0	**0.045**
Use of antibiotics in the 3 months prior to birth, yes	-0.05	-0.25; 0.16	0.652	-0.24	-0.44; -0.05	**0.016**
Intrapartum antibiotic prophylaxis (IAP)	0.03	-0.19; 0.25	0.775	0.00	-0.15; 0.14	0.987
Sex, male	-0.13	-0.33; 0.07	0.191	0.04	-0.09; 0.17	0.553
Intubation, yes*	-0.04	-0.33; 0.24	0.760			
Postnatal antibiotics, yes*	-0.14	-0.34; 0.06	0.172			
Postnatal antibiotics, days*	-0.03	-0.08; 0.01	0.119			
Chorioamnionitis, yes*	-0.01	-0.24; 0.21	0.905			
Duration of hospitalization, days*	0.00	0; 0	0.825			
Hospitalization, yes*	-0.13	-0.41; 0.15	0.351			
Probiotics, yes*	0.04	-0.17; 0.24	0.728			
Breastfeeding at date of nasal swab, yes	-0.03	-0.23; 0.18	0.796	-0.13	-0.33; 0.07	0.203
Presence of older siblings, yes	-0.22	-0.43; -0.01	**0.043**	-0.15	-0.28; -0.02	**0.020**
Study center, Basel				-0.09	-0.21; 0.04	0.185
Season at swab collection						
Winter	Ref			Ref		
Spring	-0.05	-0.34; 0.23	0.722	0.06	-0.12; 0.25	0.494
Summer	-0.09	-0.35; 0.18	0.517	0.12	-0.06; 0.31	0.175
Fall	0.02	-0.28; 0.32	0.877	0.15	-0.04; 0.33	0.121

Abbreviation: β—regression coefficient; CI—confidence interval; ref—reference
Estimates were obtained using univariable linear regression. *Due to the low number of observations these variables were not included in the regression model in full-term infants

Table E2: Univariable linear regression model of richness in preterm and full-term infants

	Preterm infants			Full-term infants		
Variables	***β***	***95% CI***	***p-value***	***β***	***95% CI***	***p-value***
Gestational age, weeks	0.00	-0.02; 0.01	0.505	0.01	-0.01; 0.03	0.486
Postmenstrual age at nasal swab, weeks	0.01	-0.02; 0.04	0.654	0.01	-0.01; 0.03	0.306
Postnatal age, weeks	0.00	0; 0	0.436	0.00	0; 0.01	0.568
Cesarean section, yes	0.02	-0.07; 0.11	0.625	-0.01	-0.07; 0.05	0.680
Smoking during pregnancy, yes	-0.07	-0.23; 0.1	0.413	-0.03	-0.17; 0.11	0.700
Antibiotics during pregnancy, yes	-0.04	-0.12; 0.04	0.300	-0.05	-0.11; 0.01	0.084
Use of antibiotics in the 3 months prior to birth, yes	-0.03	-0.11; 0.05	0.416	-0.09	-0.17; -0.01	**0.031**
Intrapartum antibiotic prophylaxis (IAP)	0.00	-0.09; 0.08	0.948	-0.02	-0.08; 0.03	0.413
Sex, male	-0.06	-0.13; 0.02	0.154	0.02	-0.03; 0.07	0.514
Intubation, yes*	0.04	-0.07; 0.15	0.449			
Postnatal antibiotics, yes*	-0.03	-0.1; 0.05	0.504			
Postnatal antibiotics, days*	-0.01	-0.02; 0.01	0.397			
Chorioamnionitis, yes*	0.01	-0.08; 0.1	0.848			
Duration of hospitalization, days*	0.00	0; 0	0.646			
Hospitalization, yes*	-0.05	-0.16; 0.06	0.341			
Probiotics, yes*	0.03	-0.05; 0.11	0.474			
Breastfeeding at date of nasal swab, yes	-0.01	-0.09; 0.07	0.858	-0.06	-0.15; 0.02	0.138
Presence of older siblings, yes	-0.05	-0.13; 0.03	0.204	-0.04	-0.09; 0.02	0.169
Study center, Basel	-0.16	-0.62; 0.29	0.471	0.00	-0.05; 0.05	0.943
Season at swab collection						
Winter	Ref			Ref		
Spring	-0.04	-0.15; 0.07	0.455	0.03	-0.05; 0.1	0.512
Summer	-0.02	-0.13; 0.08	0.628	0.06	-0.01; 0.13	0.117
Fall	0.00	-0.12; 0.11	0.963	0.07	-0.01; 0.14	0.074

Abbreviation: β—regression coefficient, CI—confidence interval; ref—reference
Estimates were obtained using univariable linear regression. *Due to the low number of observations these variables were not included in the regression model in full-term infants

Table E3: Multivariable linear regression model of Shannon indices in preterm and full-term infants

	Preterm infants (n=136)			Full-term infants (n=299)		
Variable	***β***	***95% CI***	***p-value***	***β***	***95% CI***	***p-value***
Shannon						
Use of antibiotics in the 3 months prior to birth, yes	-0.06	-0.28; 0.17	0.621	-0.24	-0.44; -0.04	**0.018**
Cesarean section, yes	0.07	-0.18; 0.33	0.569	-0.02	-0.16; 0.12	0.765
Sex, male	-0.14	-0.35; 0.07	0.176	0.03	-0.09; 0.16	0.614
Postnatal antibiotics, yes	-0.23	-0.51; 0.05	0.106			
Breastfeeding at date of nasal swab, yes	0.03	-0.19; 0.25	0.792	-0.15	-0.35; 0.05	0.136
Probiotics, yes*	0.07	-0.23; 0.36	0.659			
Season at swab collection						
Winter	Ref			Ref		
Spring	-0.06	-0.36; 0.24	0.689	0.12	-0.07; 0.3	0.218
Summer	-0.06	-0.34; 0.21	0.654	0.17	-0.01; 0.34	0.071
Fall	-0.03	-0.35; 0.29	0.841	0.18	0; 0.37	**0.048**
Presence of older siblings, yes	-0.16	-0.4; 0.07	0.169	-0.20	-0.33; -0.06	**0.004**
Postnatal age, days	0.00	0; 0.01	0.605	0.01	0; 0.02	0.202
Study center, Basel				-0.10	-0.23; 0.03	0.143

Abbreviation: β—regression coefficient; CI—confidence interval; ref—reference

Estimates were obtained using multivariable linear regression with adjustment for all covariates listed in the table for preterm and full-term infants separately. *Due to the low number of observations these variables were not included in the regression model in full-term infants

Table E4: Multivariable linear regression model of Simpson indices in preterm and full-term infants

Simpson

	Preterm infants (n=136)			Full-term infants (n=299)		
Variable	***β***	***95% CI***	***p-value***	***β***	***95% CI***	***p-value***
Use of antibiotics in the 3 months prior to birth,yes	-0.04	-0.13; 0.05	0.360	-0.10	-0.18; -0.01	**0.021**
Cesarean section, yes	0.02	-0.08; 0.12	0.672	-0.02	-0.08; 0.03	0.415
Sex, male	-0.07	-0.15; 0.02	0.113	0.02	-0.03; 0.07	0.493
Postnatal antibiotics, yes	-0.07	-0.18; 0.04	0.193			
Breastfeeding at date of nasal swab, yes	0.01	-0.07; 0.1	0.759	-0.07	-0.15; 0.01	0.089
Probiotics, yes*	0.02	-0.09; 0.14	0.684			
Season at swab collection						
Winter	Ref			Ref		
Spring	-0.04	-0.16; 0.08	0.483	0.04	-0.03; 0.12	0.282
Summer	-0.03	-0.13; 0.08	0.640	0.07	0; 0.15	0.050
Fall	-0.02	-0.15; 0.1	0.695	0.08	0.01; 0.16	**0.032**
Presence of older siblings, yes	-0.03	-0.12; 0.06	0.502	-0.05	-0.1; 0.01	0.105
Postnatal age, days	0.00	0; 0	0.427	0.00	0; 0.01	0.239
Study center, Basel				0.00	-0.05; 0.06	0.919

Abbreviation: β—regression coefficient; CI—confidence interval; ref—reference
Estimates were obtained using multivariable linear regression with adjustment for all covariates listed in the table for preterm and full-term infants separately. *Due to the low number of observations these variables were not included in the regression model in full-term infants

Table E5: Effect of exposure factors on β-diversity (Bray–Curtis distance) of preterm infants by permutational multivariate analysis of variance (PERMANOVA)

	R^2	p-value
Presence of older siblings, yes	0.017	**0.013**
Sex, male	0.006	0.549
Probiotics, yes	0.008	0.328
Use of antibiotics in the 3 months prior to birth, yes	0.007	0.400
Breastfeeding at date of nasal swab, yes	0.009	0.187
Postnatal antibiotics, yes	0.011	0.087
Cesarean section, yes	0.009	0.221
Season at swab collection*	0.028	0.113

Abbreviation: R^2—measure of group separation varying form 0: no separation to 1: total separation

*winter vs. other seasons combined

Table E6: Relative abundance at genus level in preterm infants stratified by presence of older siblings

	no older siblings (n=92)		presence of older siblings (n=44)			
**Genus**	**mean (sd)**	**median (Q1; Q3)**	**mean (sd)**	**median (Q1; Q3)**	**p-value**	**p-value$_{adj}$**
Other	0.25 (2.07)	0 (0 ;0)	0.23 (3.96)	0 (0; 0)	5.4×10^{-8}	$\mathbf{4.3 \times 10^{-7}}$
Acinetobacter	4.77 (12.43)	0.55 (0.13; 2.37)	1.3 (4.62)	0.1 (0.04; 0.48)	3.2×10^{-4}	$\mathbf{1.3 \times 10^{-3}}$
Corynebacterium	30.89 (31.98)	18.31 (2.1; 49.6)	17.11 (25.71)	4.04 (0.54; 20.78)	7.4×10^{-3}	$\mathbf{2.0 \times 10^{-2}}$
Moraxella	4.78 (18.18)	0 (0; 0)	23.78 (33.59)	0 (0; 53.13)	1.0×10^{-2}	$\mathbf{2.0 \times 10^{-2}}$
Dolosigranulum	8.1 (18.9)	0 (0; 0.1)	9.97 (20.11)	0.15 (0; 7.96)	3.8×10^{-2}	6.2×10^{-2}
Haemophilus	0.07 (0.35)	0 (0; 0.01)	5.77 (16.45)	0 (0; 0.12)	9.7×10^{-2}	1.3×10^{-1}
Staphylococcus	33.92 (38.14)	13.27 (1.11; 79.07)	31.4 (38.27)	13.54 (0.04; 69.38)	2.2×10^{-1}	2.5×10^{-1}
Streptococcus	9.9 (16.28)	1.95 (0.3; 9.53)	6.56 (12.95)	0.91 (0.2; 6.06)	2.5×10^{-1}	2.5×10^{-1}

* Low abundant genera with a mean of relative abundance <0.01 was combined in the group "other".

P-value was calculated using the Mann–Whitney test. P-value$_{adj}$ was obtained using Benjamini–Hochberg correction.

Table E8: Population characteristics of preterm infants stratified by probiotic use

	**No Probiotics (N=81)**	**Probiotics (N=55)**	**P-value**
Sex, male, n (%)	44 (54.3%)	29 (52.7%)	0.994
Gestational age, weeks, mean (SD)	34.1 (1.77)	29.9 (2.24)	**<0.001**
Birth weight, z-score, mean (SD)	-0.317 (0.737)	-0.357 (0.766)	0.763
Birth weight, g, mean (SD)	2130 (494)	1270 (315)	**<0.001**
Postnatal age, days, mean (SD)	73.6 (16.4)	103 (18.3)	**<0.001**
Postmenstrual age at nasal swab, weeks, mean (SD)	44.6 (1.25)	44.6 (1.30)	0.872
Season of swab collection			
Winter	17 (21.0%)	22 (40.0%)	0.079
Spring	22 (27.2%)	8 (14.5%)	
Summer	26 (32.1%)	16 (29.1%)	
Fall	16 (19.8%)	9 (16.4%)	
Presence of older siblings, yes	32 (39.5%)	12 (21.8%)	**0.048**
Cesarean section, yes n (%)	56 (69.1%)	48 (87.3%)	**0.025**
Breastfeeding at date of nasal swab, yes n (%)	46 (56.8%)	32 (58.2%)	1
Chorioamnionitis, yes	14 (17.3%)	23 (41.8%)	**0.004**
Antibiotics during pregnancy, yes n (%)	34 (42.0%)	26 (47.3%)	0.664
Antibiotics during last 3 months before birth, yes n (%)	30 (37.0%)	21 (38.2%)	1
Postnatal antibiotics for neonatal infection, yes n (%)	14 (17.3%)	42 (76.4%)	**<0.001**
Hospitalization, yes n (%)	60 (74.1%)	55 (100%)	**<0.001**
Length of hospitalization, days, mean (SD)	18.3 (16.9)	55.9 (24.3)	**<0.001**
Intubation, yes n (%)	4 (4.9%)	16 (29.1%)	**<0.001**
Postnatal antibiotics for neonatal infection, days, mean (SD)	0.580 (1.40)	3.22 (2.45)	**<0.001**

P-values were obtained using Student's t-test, the Mann-Whitney test, and Fisher's exact test, as appropriate

Table E9: Multivariable linear regression model of Shannon diversity and Simpson indices in all infants

	Shannon			Simpson		
	β	***95% CI***	***p-value***	***β***	***95% CI***	***p-value***
Term birth, yes	0.01	-0.23; 0.26	0.920	0.03	-0.07; 0.13	0.516
Use of antibiotics in the 3 months prior to birth,yes	-0.11	-0.26; 0.03	0.122	-0.05	-0.11; 0.01	0.087
Cesarean section, yes	0.00	-0.12; 0.12	0.984	-0.02	-0.06; 0.03	0.549
Sex, male	-0.02	-0.13; 0.09	0.686	-0.01	-0.05; 0.04	0.812
Postnatal antibiotics, yes	-0.25	-0.48; -0.01	**0.044**	-0.08	-0.18; 0.01	0.087
Breastfeeding at date of nasal swab, yes	-0.07	-0.21; 0.07	0.331	-0.03	-0.09; 0.02	0.239
Probiotics, yes	0.12	-0.14; 0.38	0.355	0.05	-0.05; 0.16	0.306
Season at swab collection						
Winter	Ref			Ref		
Spring	0.04	-0.11; 0.2	0.571	0.01	-0.05; 0.07	0.749
Summer	0.07	-0.08; 0.22	0.337	0.04	-0.02; 0.1	0.239
Fall	0.11	-0.05; 0.27	0.172	0.05	-0.01; 0.11	0.129
Presence of older siblings, yes	-0.19	-0.3; -0.07	**0.001**	-0.04	-0.09; 0	0.080
Postnatal age, days	0.00	0; 0.01	0.459	0.00	0; 0	0.334
Study center, Basel	-0.12	-0.25; 0.02	0.088	0.00	-0.06; 0.05	0.856

Abbreviation: β—regression coefficient; CI—confidence interval
Estimates were obtained using multivariable linear regression with adjustment for all covariates listed in the table

Table E10: Effect of exposure factors on β-diversity (Bray–Curtis distance) in full-term infants and the overall population preterm and full-term infants using permutational multivariate analysis of variance (PERMANOVA)

	**R^2**	**p-value**
**Full-term Infants**		
Presence of older siblings, yes	0.033	**0.001**
Sex, male	0.004	0.244
Use of antibiotics in the 3 months prior to birth, yes	0.005	0.076
Breastfeeding at date of nasal swab, yes	0.003	0.505
Cesarean section, yes	0.007	**0.015**
Study center, Basel	0.006	**0.029**
Season at swab collection*	0.014	**0.042**
**Overall Population**		
Term birth	0.014	**0.001**
Presence of older siblings, yes	0.019	**0.001**
Sex, male	0.0031	0.141
Probiotics, yes	0.002	0.485
Use of antibiotics in the 3 months prior to birth, yes	0.003	0.192
Breastfeeding at date of nasal swab, yes	0.003	0.157
Postnatal antibiotics, yes	0.003	0.116
Cesarean section, yes	0.007	**0.002**
Study center, Basel	0.005	**0.020**
Season at swab collection*	0.012	**0.006**

Abbreviation: R^2—measure of group separation varying form 0: no separation to 1: total separation

*winter vs. other seasons combined

Table E11: Relative abundance at phylum level in preterm and full-term infants

	preterm (n=136)		full-term (n=299)			
**Phylum**	**mean (sd)**	**median (Q1-Q3)**	**mean (sd)**	**median (Q1-Q3)**	**p-value**	**p-value$_{adj}$**
Proteobacteria	0.23 (0.31)	0.05 (0.01; 0.34)	0.34 (0.37)	0.14 (0.02; 0.71)	1.8×10^{-4}	**7.3×10^{-4}**
Firmicutes	0.51 (0.34)	0.52 (0.18; 0.86)	0.41 (0.33)	0.37 (0.1; 0.69)	4.6×10^{-2}	9.2×10^{-2}
Actinobacteriota	0.26 (0.3)	0.13 (0.02; 0.45)	0.25 (0.29)	0.11 (0.02; 0.42)	2.5×10^{-1}	3.3×10^{-1}

P-value was calculated using the Mann–Whitney test. P-value$_{adj}$ was obtained using Benjamini–Hochberg correction.

Table E12: Relative abundance at genus level in preterm and full-term infants

Genus	preterm (n=136) mean (sd)	preterm (n=136) median (Q1-Q3)	full-term (n=299) mean (sd)	full-term (n=299) median (Q1-Q3)	p-value	p-value$_{adj}$
Staphylococcus	33.11 (38.06)	13.36 (0.91; 76.3)	18.46 (30.13)	1.42 (0.09; 24.39)	5.9×10^{-6}	$\mathbf{4.7 \times 10^{-5}}$
Moraxella	10.93 (25.72)	0 (0; 0.03)	25.26 (36.88)	0 (0; 56.3)	3.7×10^{-5}	$\mathbf{1.5 \times 10^{-4}}$
Dolosigranulum	8.7 (19.24)	0 (0; 5.24)	13.19 (21.12)	0.72 (0; 20.49)	6.3×10^{-5}	$\mathbf{1.7 \times 10^{-4}}$
Haemophilus	1.91 (9.66)	0 (0; 0.01)	2.35 (12.16)	0 (0; 0.05)	2.0×10^{-2}	$\mathbf{3.9 \times 10^{-2}}$
Acinetobacter	3.65 (10.66)	0.37 (0.07; 1.66)	3.25 (7.69)	0.38 (0.09; 2.74)	4.7×10^{-1}	7.5×10^{-1}
Streptococcus	8.82 (15.31)	1.53 (0.25; 8.84)	8.97 (17.29)	1.65 (0.26; 8.14)	7.2×10^{-1}	8.5×10^{-1}
Other*	0.25 (2.82)	0 (0; 0)	0.16 (2.34)	0 (0; 0)	7.4×10^{-1}	8.5×10^{-1}
Corynebacterium	26.43 (30.69)	13.31 (1.42; 45.48)	25.09 (29.15)	11.33 (1.41; 42.31)	9.8×10^{-1}	9.8×10^{-1}

* Low abundant genera with a mean of relative abundance <0.01 was combined in the group "other".

P-value was calculated using the Mann–Whitney test. P-value$_{adj}$ was obtained using Benjamini–Hochberg correction.

FIGURES

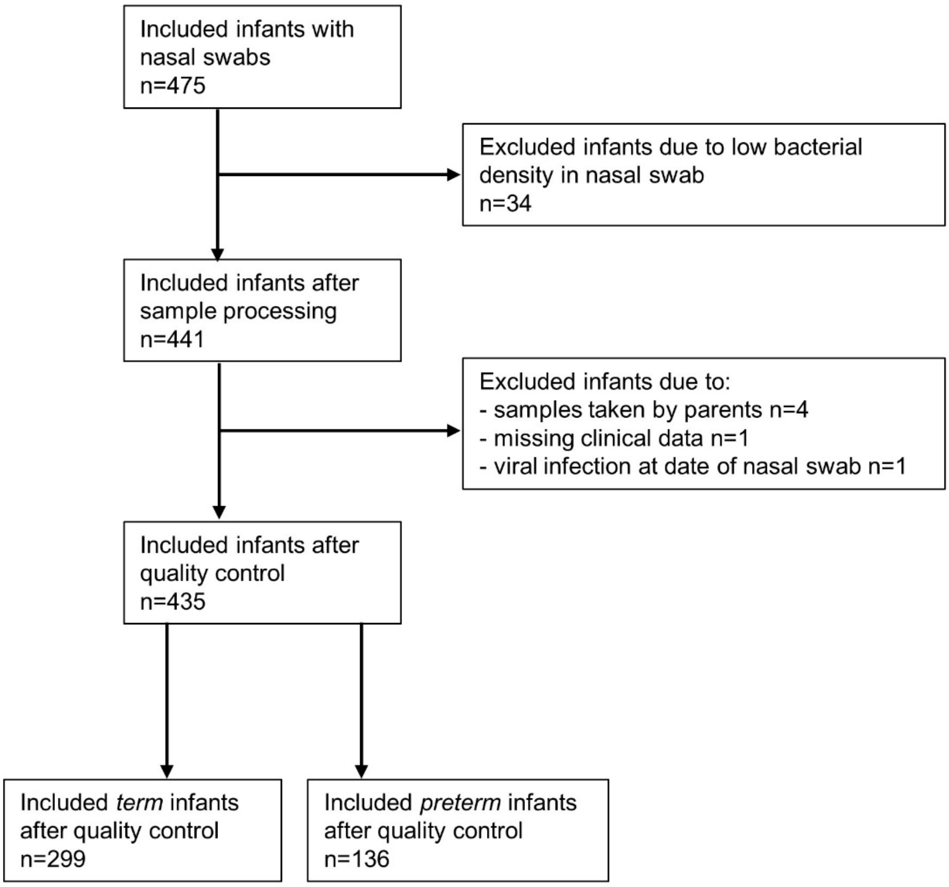

Figure E1: Study flowchart

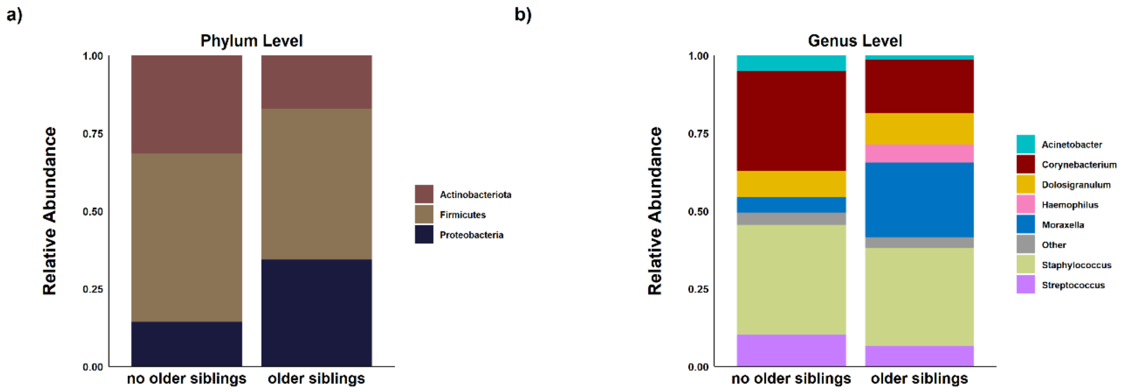

Figure E2: Mean of relative abundance of the phyla (**a**) and genera (**b**) identified in groups of preterm infants with and without older siblings. The low-abundance phylum *Bacteroidota* was not included due to its mean abundance being less than 0.01. Low abundant genera with mean of relative abundance <0.01 was combined in the “other” group.

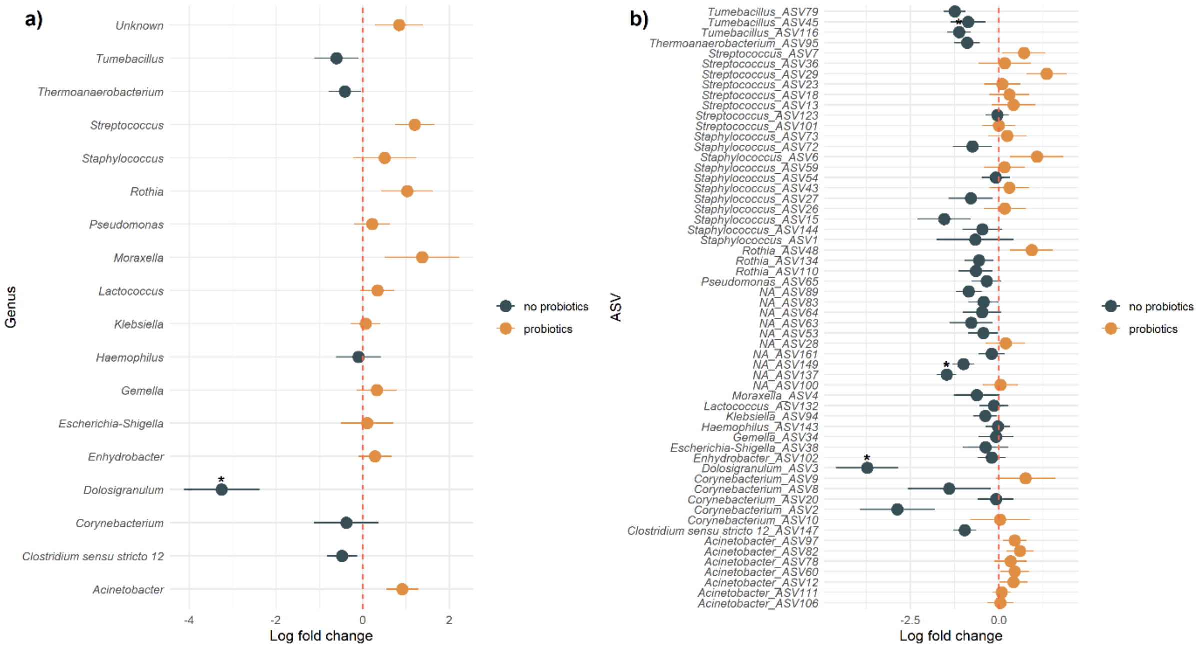

Figure E3: Differentially abundant genera and ASVs in nasal samples of preterm infants with and without probiotic use. Differential abundance testing was done using ANCOM-BC accounting for postnatal age, presence of older siblings, season, use of antibiotics in the 3 months prior to birth, postnatal antibiotic use, and breastfeeding at swab collection. Taxa that feature in less than 10% of all samples were removed from analysis. Points show the log fold change as given by ANCOM-BC, error bars denote the standard error at the genus (**a**) and ASV (**b**) level of taxa in preterm and full-term infants. Asterisks indicate significant p-values following Benjamini–Hochberg adjustment for multiple testing.

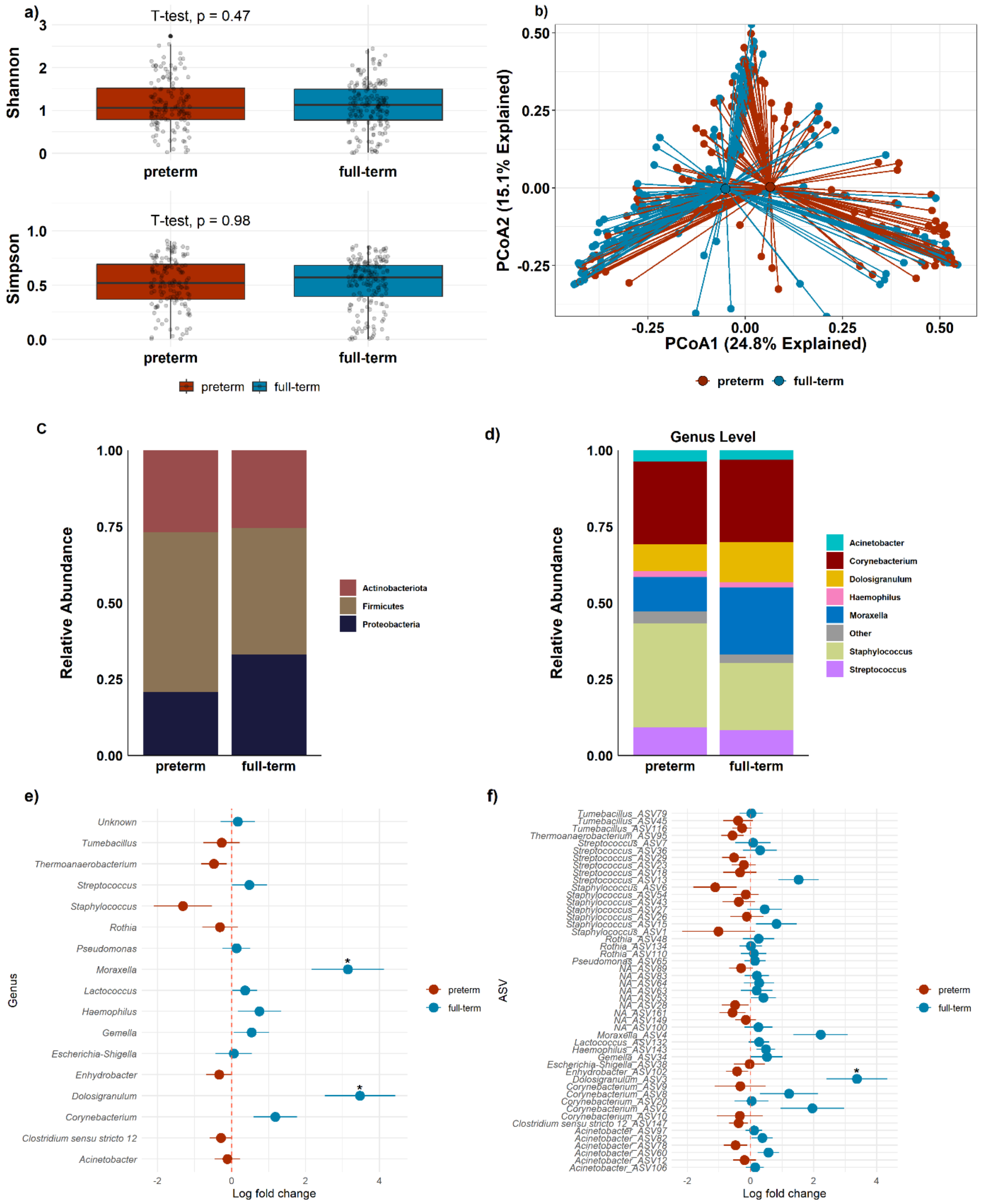

Figure E4: The nasal microbiota in preterm and full-term infants only from Basel (n=304). (**a**) Shannon and Simpson diversity in nasal samples of preterm and full-term infants. (**b**) Principal coordinate analysis (PcoA) of nasal samples of preterm and full-term infants (PERMANOVA p-value=0.001, F=3.7, R^2=0.012 with adjustment for presence of older siblings, sex, use of antibiotics in the 3 months prior to birth, postnatal antibiotics use, probiotics, mode of delivery, season of swab collection, and any breastfeeding at swab collection). Bar plots show average relative abundances at the phylum (**c**) and genus (**d**) level of the most abundant taxa in preterm and full-term infants. The low-abundance phylum Bacteroidota was not included due to its mean abundance being less than 0.01. Low abundant

genera with mean of relative abundance <0.01 was combined in the “other” group. Differentially abundant genera **(e)** and ASVs **(f)** in nasal samples of preterm and full-term infants. Differential abundance testing was done using ANCOM-BC accounting for postnatal age, presence of older siblings, season, use of antibiotics in the 3 months prior to birth, postnatal antibiotic use, breastfeeding at swab collection, and study center. Points show the log fold change as given by ANCOM-BC, error bars denote the standard error. Asterisks indicate significant p-values following Benjamini–Hochberg adjustment for multiple testing.

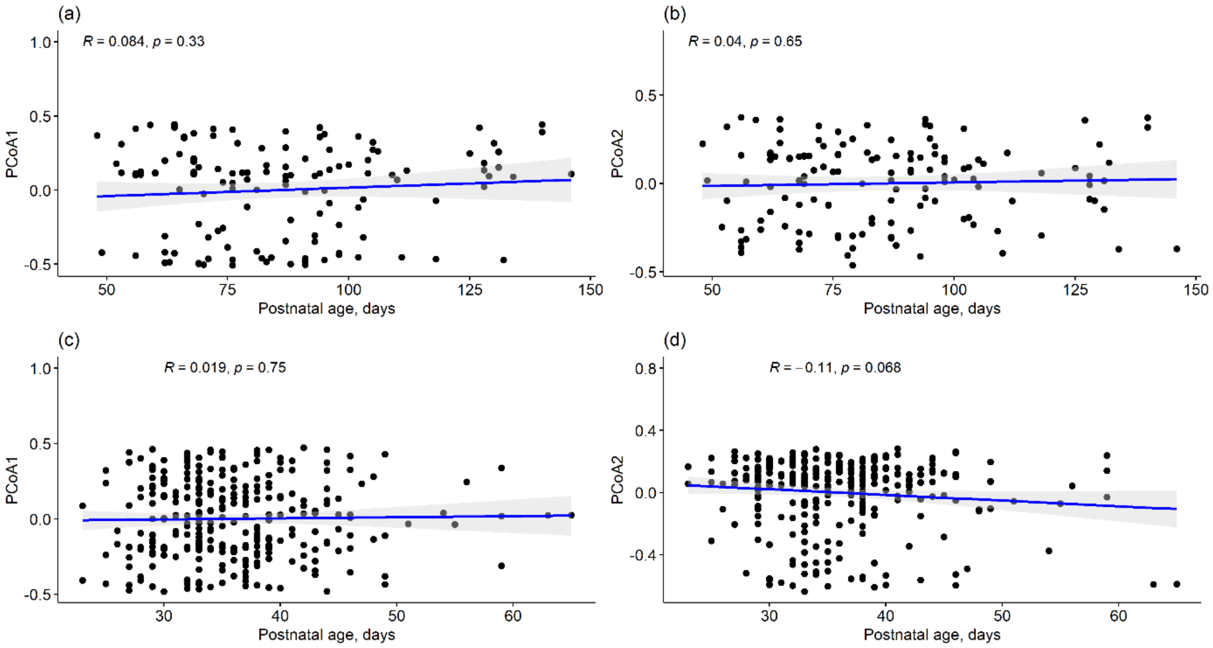

Figure E5: Scatter plot illustrating the relationship between postnatal age and the first two principal components (PCs) for β-diversity in preterm (**a**, **b**) and full-term infants (**c**, **d**). Each plot includes the Pearson correlation coefficient with p-value.
